# Intensive cycles of neoadjuvant camrelizumab combined with chemotherapy in locally advanced esophageal squamous cell carcinoma: a single-arm, phase II trial

**DOI:** 10.1186/s12967-023-04273-6

**Published:** 2023-06-24

**Authors:** Guozhen Yang, Xiaodong Su, Yuanheng Huang, Guangyu Luo, Zhiqiang Wang, Peiqiang Cai, Yating Zheng, Ting Bei, Mengli Huang, Yuezong Bai, Haoqiang He, Jin Xiang, Muyan Cai, Jiudi Zhong, Qiyu Guo, Xu Zhang

**Affiliations:** 1 Department of Thoracic Oncology, Sun Yat-Sen University Cancer Center, Guangzhou, China; 2 Guangdong Esophageal Cancer Institute, Guangzhou, China; 3 State Key Laboratory of Oncology in South China, Collaborative Innovation Center of Cancer Medicine, Guangzhou, China; 4 Department of Endoscopy, Sun Yat-Sen University Cancer Center, Guangzhou, China; 5 Department of Medical Oncology, Sun Yat-Sen University Cancer Center, Guangzhou, China; 6 Department of Medical Imaging and Interventional Radiology, Sun Yat-Sen University Cancer Center, Guangzhou, China; 7 Medical Affairs, 3D Medicines, Inc, Shanghai, China; 8 Department of Pathology, Sun Yat-Sen University Cancer Center, Guangzhou, China

**Keywords:** Neoadjuvant, Immunotherapy, Chemotherapy, ESCC

## Abstract

**Background:**

Two cycles of neoadjuvant PD-1 blockade plus chemotherapy induced favorable pathological response and tolerant toxicity in patients with locally advanced esophageal squamous cell carcinoma (ESCC). However, approximately 25% of patients relapsed within 1 year after surgery, indicating that a short course of treatment may not be sufficient. Therefore, exploring the effects of intensive treatment is needed for optimal clinical outcomes.

**Methods:**

Locally advanced ESCC patients were administered three cycles of camrelizumab plus nab-paclitaxel and capecitabine, followed by thoracoscopic esophagectomy. The primary endpoint was pathologic response. Secondary endpoints included safety, feasibility, radiologic response, survival outcomes, and immunologic/genomic correlates of efficacy.

**Results:**

Forty-seven patients were enrolled in the study. Forty-two patients received surgery, and R0 resection was achieved in all cases. The complete and major pathological response rates were 33.3% and 64.3%, respectively, and the objective response rate was 80.0%. Three cycles of treatment significantly improved T down-staging compared to two cycles (*P* = 0.03). The most common treatment-related adverse events were grades 1–2, and no surgical delay was reported. With a median follow-up of 24.3 months, the 1-year disease-free survival and overall survival rates were both 97.6%, and the 2-year disease-free survival and overall survival rates were 92.3% and 97.6%, respectively. Three patients experienced disease recurrence or metastasis ranging from 12.5 to 25.8 months after surgery, and one patient died 6 months after surgery due to cardiovascular disease. Neither programmed death-ligand 1 expression nor tumor mutational burden was associated with pathological response. An increased infiltration of CD56^*dim*^ natural killer cells in the pretreatment tumor was correlated with better pathological response in the primary tumor.

**Conclusions:**

It seems probable that intensive cycles of neoadjuvant camrelizumab plus nab-paclitaxel and capecitabine increased tumor regression and improved survival outcomes. Randomized controlled trials with larger sample sizes and longer follow-up periods are needed to validate these findings.

*Trial registration* Chinese Clinical Trial Registry, ChiCTR2000029807, Registered February 14, 2020, https://www.chictr.org.cn/showproj.aspx?proj=49459.

**Supplementary Information:**

The online version contains supplementary material available at 10.1186/s12967-023-04273-6.

## Background

Esophageal cancer (EC) is the seventh most common malignant tumor and the sixth leading cause of cancer-related mortality worldwide [1]. In China, approximately 90% of EC cases are squamous cell carcinoma [2, 3]. Esophagectomy plays a primary role in treating locally advanced esophageal squamous cell carcinoma (ESCC). However, 45% of the patients with surgery alone experience local recurrence or distant metastasis within 5 years after surgery [4–6].

Neoadjuvant therapy could increase the rate of R0 resection and improve survival compared with surgery alone [6]. Based on the results of the NEOCRTEC5010 trial [6], chemoradiotherapy followed by surgery has been recommended as the standard treatment for locally advanced ESCC. However, radiotherapy can increase perioperative complications and mortality. Additionally, despite the high R0 resection rate with chemoradiotherapy, about 15% of ESCC patients still suffer from local recurrence within 5 years after surgery, and that rate of distant metastasis reaches 30% [6, 7]. Therefore, novel strategies are needed to achieve better safety profiles and optimal survival outcomes.

As a newcomer to cancer treatment, programmed cell death 1 (PD-1) blockade-based immunotherapy exploits a strategy based on immune evasion mechanisms to restore antitumor immunity. Camrelizumab is a humanized high-affinity IgG4-kappa anti-PD-1 monoclonal antibody that has demonstrated efficacy and safety in patients with advanced ESCC [8, 9]. The randomized phase III ESCORT-1^st^ study reported that the addition of camrelizumab to chemotherapy improved overall survival (OS) and progression-free survival (PFS) compared with chemotherapy alone, and it has been approved to treat unresectable advanced ESCC with camrelizumab plus chemotherapy in China [10]. In addition, neoadjuvant use of PD-1 blockade in combination with chemotherapy has also shown favorable antitumor efficacy in several malignancies, including lung [11, 12] and colorectal cancers [13, 14]. However, the combination of PD-1 blockade with chemotherapy in locally advanced ESCC has not been well determined.

To date, a few clinical trials have reported that neoadjuvant immunochemotherapy of PD-(L)1 blockade induced a favorable pathological response and tolerant toxicity in patients with locally advanced ESCC [15, 16]. However, all these studies were small cohort studies with only two cycles. Theoretically, immunochemotherapy has a huge potential to induce long-term tumor regression, eradicate micrometastases, and even cure locally advanced ESCC [17–19]. According to previous data of locally advanced ESCC who received two cycles of PD-1 blockade plus chemotherapy, at a median follow-up of 13 months, recurrence still occurred in approximately 25% of patients, indicating a short course of treatment may not be sufficient [16]. Indeed, a retrospective study based on real-world data reported that patients with locally advanced ESCC received varying cycles of neoadjuvant immunochemotherapy, with the majority receiving 2–4 cycles. However, the relationship between treatment cycles and pathological responses was not investigated [20]. Meanwhile, a real-world retrospective study in lung cancer has shown that three and four cycles of neoadjuvant immunochemotherapy were prone to higher major pathological response (MPR) rates than two cycles [17]. Given the encouraging efficacy and acceptable safety of PD-(L)1 blockade plus chemotherapy in solid tumors, intensive treatment deserves to be explored for optimal clinical outcomes.

In our previous retrospective study [21], intensive cycles of camrelizumab plus chemotherapy before surgery exhibited promising efficacy without increasing complications in locally advanced ESCC. Therefore, we further performed this phase II trial to evaluate the efficacy and safety of intensive treatment in locally advanced ESCC. Computerized tomography (CT) and safety assessment were conducted before the initiation of treatment and after the second and third courses of neoadjuvant immunochemotherapy to compare the efficacy and safety of two and three treatment cycles. In addition, little is known about biomarkers predicting the efficacy of neoadjuvant immunochemotherapy, which have also been explored in this study.

## Methods

### Participants

In this single-center, single-arm, phase II trial, camrelizumab was combined with chemotherapy followed by surgery for locally advanced ESCC. Inclusion criteria were (1) stage II or III locally advanced resectable ESCC diagnosed before enrollment (2) no distant organ metastases or cervical lymph node metastases prior to enrollment (3) no secondary primary tumors (4) an Eastern Cooperative Oncology Group (ECOG) performance status score 0 or 1 (5) no prior exposure to anticancer therapy, including chemotherapy, radiotherapy, targeted therapy, and immunotherapy.

### Procedure

Participants were administered three cycles of chemotherapy and PD-1 blockade. For each cycle of treatment, patients were intravenously administered a flat dose of camrelizumab (200 mg) along with a single dose of nab-paclitaxel (260 mg/m^2^) on day 1, and capecitabine was orally administered twice daily (1250 mg/m^2^) on days 1 through 14. The regimen was repeated every 3 weeks (Additional file 1: Fig. S1). A prophylactic dose of granulocyte colony-stimulating factor (G-CSF) was administered on day 4 of each cycle. The following tests were performed at baseline, two and three times after the neoadjuvant treatment cycles: contrasted-enhanced thoracic/abdominal CT, endoscopic ultrasonography (EUS), and cervical/subclavicular ultrasonography. Radiographic responses of primary tumors were evaluated using CT scan images acquired before and after two and three cycles of neoadjuvant treatment according to Response Evaluation Criteria in Solid Tumors version (RECIST) 1.1 [22]. All imaging data were reviewed by two independent radiologists. Treatment-related adverse events (TRAEs) were reported according to the National Cancer Institute Common Terminology Criteria for Adverse Events (NCI-CTCAE), version 5.0, at each visit [23].

In approximately four to six weeks after the last course of neoadjuvant therapy, a thoracoscopy esophagectomy was performed with cervical esophagogastric anastomosis and total dissection of two-field lymph nodes (LNs). The removal of lymph nodes included recurrent laryngeal nerve nodes, subcarinal nodes, paraesophageal nodes, pulmonary ligament nodes, cardia nodes, left gastric artery nodes, and lesser curvature nodes. Surgical sections were stained with hematoxylin and eosin (H&E), and pathological regression was assessed by two independent pathologists. Complete pathological response (pCR) was defined as the absence of residual invasion disease. Tumors with ≤ 10% residual viable tumor cells were considered as obtaining an MPR.

After surgery, follow-up was conducted every 3 months in the first year, every 6 months for the second and third years, and every 12 months thereafter. Overall survival (OS) was defined as the time between the surgery and the end of follow-up or death. Disease-free survival (DFS) was calculated from the surgery date to the end of follow-up or the date of the first recurrence.

### Outcome

The primary endpoint of the study was pCR. The secondary endpoints included safety, feasibility, MPR, radiologic response, DFS, and OS.

### Exploratory analysis

Pretreatment tumor biopsy was obtained using EUS for biomarker analysis, including programmed cell death-ligand 1 (PD-L1) expression, tumor mutational burden (TMB), and tumor immune microenvironment (TIME). PD-L1 expression was assessed using the PD-L1 IHC 22C3 pharmDx assay (Agilent Technologies). The combined positive score (CPS) was used to define PD-L1 expression, which was determined by dividing the number of PD-L1-positive tumor and immune cells by the total number of viable tumor cells and multiplying by 100. Next-generation sequencing (NGS) was performed using whole-exome sequencing or a 733-gene panel (3D Medicines Inc.). As defined, the TMB was the number of somatic single nucleotide variations (SNVs) and insertions/deletions (indels) per megabase of coding genome sequenced. Synonymous and non-synonymous mutations, stop gains/losses, and splicing variants were all considered SNVs. Indels included both frameshift and non-frameshift insertions and deletions. Non-coding alterations were excluded from the calculation of TMB. TIME was evaluated using multiplex immunofluorescence (mIF) staining. The quantities of CD8^+^ T cells, tumor-associated macrophages (TAMs), and natural killer (NK) cells were expressed as the number of stained cells per square millimeter. Posttreatment tissue was also collected and subjected to mIF to analyze the change in the TIME after neoadjuvant immunotherapy. Besides, the posttreatment tissues were also submitted to H&E staining and immunostaining for CD3 and CD20 to analyze the tertiary lymphoid structures (TLSs).

### Statistical analyses

This study applied superiority designs with the primary endpoint of pCR. According to previous studies, the pCR rate of chemotherapy is hypothesized to be 15% [24, 25]. With the consideration of a dropout rate of 10%, a total of 47 patients would need to be enrolled to provide 80% power to detect a pCR of 34% at a one-sided 5% alpha level. Continuous variables were compared using the Mann–Whitney U test, and categorical variables were compared using the chi-square or Fisher exact test, as appropriate. All reported *P* values were two-tailed. A *P* value of < 0.05 was considered statistically significant. Survival curves were estimated using the Kaplan–Meier method. All analyses and graph generation were performed using R 3.6.0.

## Results

### Baseline characteristics

Forty-seven patients were enrolled between May 2020 and December 2021 at Sun Yat-sen University Cancer Center. The patient characteristics for the entire cohort are presented in Table 1. The median age of the cohort was 58 years (range: 44–70 years). Most were male (80.9%), had moderately-differentiated tumors (63.8%), were former smokers (70.2%), and with ECOG status score of 0 at enrollment (87.2%). Eleven and 36 patients were diagnosed as stage II and III, respectively, according to the TNM staging system. There were 2, 26, and 19 cases of lesions in the esophagus' upper, middle and lower segments, respectively.

**Table 1:** Baseline characteristics of the patients

Characteristics	No. (%)
Age at diagnosis, years	
Mean ± S.D^†^	58.8 ± 7.1
Median (range)	58 (44–70)
Sex, n (%)	
Male	38 (80.9)
Female	9 (19.1)
History of smoking, n (%)	
Former or current	33 (70.2)
Never	14 (29.8)
Site of primary tumor, n (%)	
Upper thoracic	2 (4.3%)
Middle thoracic	26 (55.3)
Lower thoracic	19 (40.4)
Histologic grade, n (%)	
Well-differentiated	5 (10.7)
Moderately differentiated	30 (63.8)
Poorly differentiated	12 (25.5)
Clinical T stage, n (%)	
T1	0 (0)
T2	12 (25.5)
T3	35 (74.5)
T4	0 (0)
Clinical N state, n (%)	
N0	3 (6.4)
N1	17 (36.2)
N2	27 (57.4)
Tumor stage^‡^, n (%)	
II	11 (23.4)
III	36 (76.6)
ECOG score, n (%)	
0	41 (87.2)
1	6 (12.8)

^†^Standard deviation

^‡^Tumor stage was evaluated following the American Joint Committee on Cancer's (AJCC) Staging Manual, 7th edition; ECOG score, Eastern Cooperative Oncology Group (ECOG) performance status (PS) score

### Treatment exposure

Of the 47 patients, 45 (95.7%) received three cycles of immunotherapy combined with chemotherapy, and two patients (4.3%) discontinued treatment after the second cycle for immune-related myocarditis (n = 1) and informed consent withdrawal (n = 1). Forty-two patients (89.4%) completed surgery as planned. The reasons for not undergoing surgery included patient refusal (n = 4) and immune-related myocarditis (n = 1).

### Safety

Neoadjuvant camrelizumab plus nab-paclitaxel and capecitabine did not cause any previously unreported TRAEs (Table 2). All patients administered neoadjuvant treatment had at least one adverse event, and most of the TRAEs were grade 1–2. The most common grade 1–2 TRAEs were alopecia (32/68.1%), reactive cutaneous capillary endothelial proliferation (RCCEP) (28/59.6%), fatigue (25/53.2%), anemia (24/51.1%), muscle soreness (20/42.6%), numbness of limbs (19/40.4%), and increased alanine transaminase (11/23.4%). Leukopenia occurred in three (6.4%) patients. Four (8.5%) patients experienced grade 3–4 adverse events, including fatigue (n = 1), limb numbness (n = 1), anemia (n = 1), and myocarditis (n = 1). No grade 5 TRAEs or treatment-related mortality were documented.

**Table 2:** Summary of treatment-related adverse events

All events	No. of patients (%)		
	Total	Grade 1–2	Grade 3–4
Alopecia	32 (68.1)	32 (68.1)	0 (0)
Reactive cutaneous capillary endothelial proliferation	28 (59.6)	28 (59.6)	0 (0)
Fatigue	26 (55.3)	25 (53.2)	1 (2.1)
Anemia	25 (53.2)	24 (51.1)	1 (2.1)
Muscle soreness	20 (42.6)	20 (42.6)	0 (0)
Limb numbness	20 (42.6)	19 (40.4)	1 (2.1)
Increased alanine transaminase	11 (23.4)	11 (23.4)	0 (0)
Constipation	8 (17.0)	8 (17.0)	0 (0)
Diarrhea	4 (8.5)	4 (8.5)	0 (0)
Immune-related hyperthyroidism	4 (8.5)	4 (8.5)	0 (0)
Leukopenia	3 (6.4)	3 (6.4)	0 (0)
Vomiting	2 (4.3)	2 (4.3)	0 (0)
Nausea	2 (4.3)	2 (4.3)	0 (0)
Immune-related hypothyroidism	2 (4.3)	2 (4.3)	0 (0)
Thrombocytopenia	2 (4.3)	2 (4.3)	0 (0)
Immune-related myocarditis	1 (2.1)	0 (0)	1 (2.1)
Cough	0 (0)	0 (0)	0 (0)
Immune-related pneumonia	0 (0)	0 (0)	0 (0)
Immune-related hepatitis	0 (0)	0 (0)	0 (0)
Immune-related nephritis	0 (0)	0 (0)	0 (0)

All adverse events were reported according to the National Cancer Institute Common Terminology Criteria for Adverse Events, version 5.0

### Surgery

Among 42 patients who underwent surgery, the mean time from the last dose of neoadjuvant therapy to surgery was 4.3 ± 1.0 weeks. R0 resection was completed in all cases, which took 255.3 ± 8.69 min on average. The intraoperative bleeding volume was 145.4 ± 52.8 ml. The mean number of lymph node dissections was 47.7 ± 2.9, and positive lymph nodes were observed in 11 (26.2%) patients. Eight (19.0%) and 1 (2.4%) patients experienced anastomotic leakage and chylothorax, respectively. The median ICU stay was one day (range, 1–7), and the median postoperative hospital stay was 15 days (range, 9–95). No postoperative immune-related adverse events or death occurred within 90 days after surgery (Additional file 1: Table S1).

### Radiography and pathological responses

**Fig. 1:**
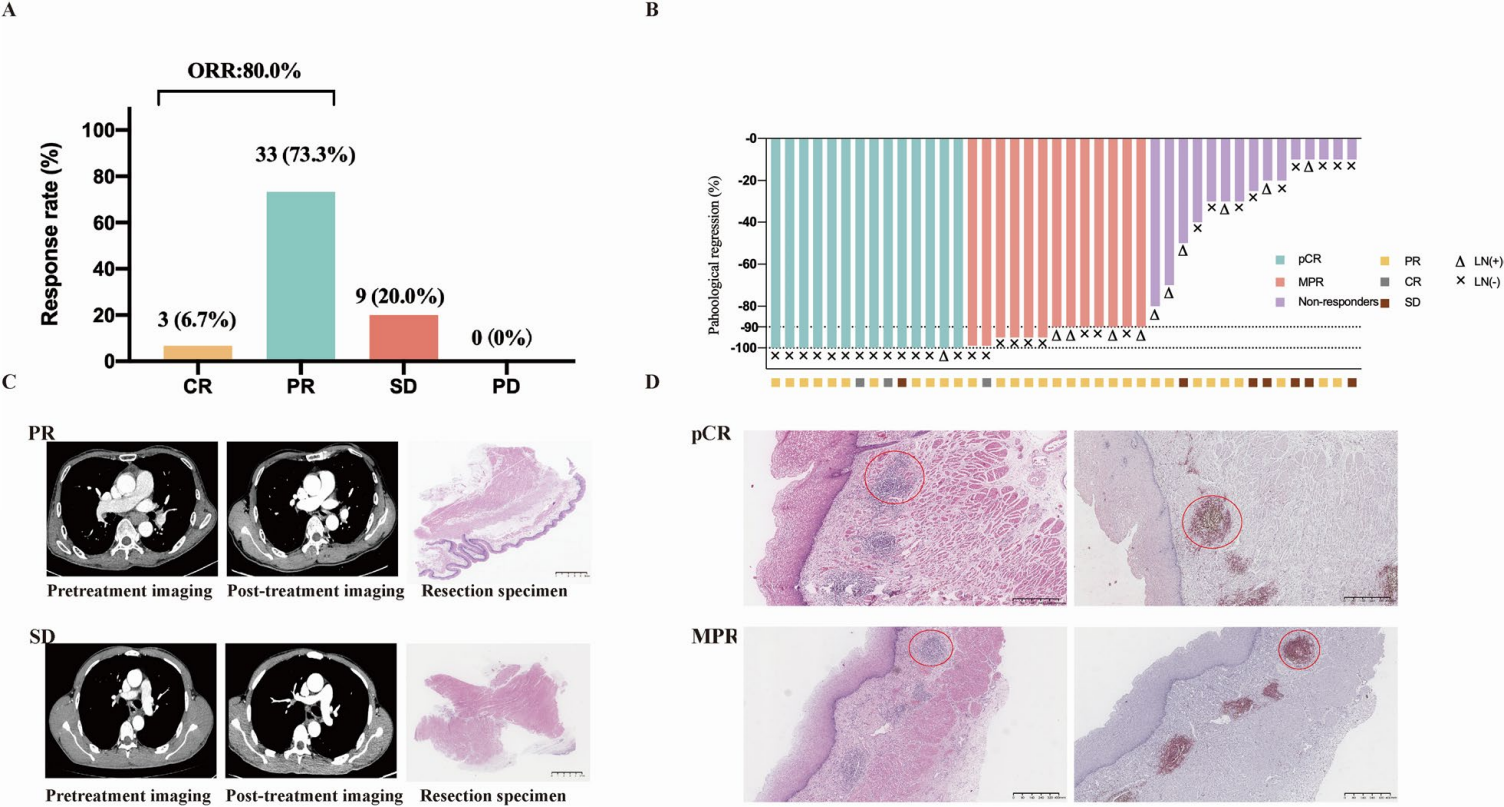
Clinical and pathological responses to neoadjuvant camrelizumab combined with chemotherapy. **A** Response assessment with CT according to RECIST 1.1. **B** Pathological tumor regression in the resected primary tumor. The presence and absence of lymph node (LN) metastasis in the resection specimen and preoperative radiologic response are annotated for each patient. **C** Representative CT images and hematoxylin and eosin-stained sections of tumor tissue obtained before neoadjuvant therapy and after surgery from a patient with PR (the upper row) and a patient with SD (the lower row). These two patients were representatives of responders and non-responders, respectively. **D** Pathological characteristics of resection specimens collected after surgery from a patient with pCR and a patient with MPR. The red circle indicates tertiary lymphoid structures. Tertiary lymphoid structures were visualized by hematoxylin and eosin-staining and immunostaining for CD3 (brown) and CD20 (red). CR: complete response; PR: partial response; SD: stable disease; PD: progressive disease; ORR: objective response rate. pCR: complete pathological response; MPR: major pathological response; CT: computed tomography. Primary tumors with more than 10% residual viable tumor cells were considered as non-responders

After three cycles of neoadjuvant treatment, three patients achieved complete response (CR), 33 had a partial response (PR), and nine had stable disease (SD), according to RECIST 1.1. No patients developed progressive disease (PD). The objective response rate (ORR) was 80.0% (95% confidence interval [CI], 65.4–90.4%) (Fig. 1A).

**Fig. 2:**
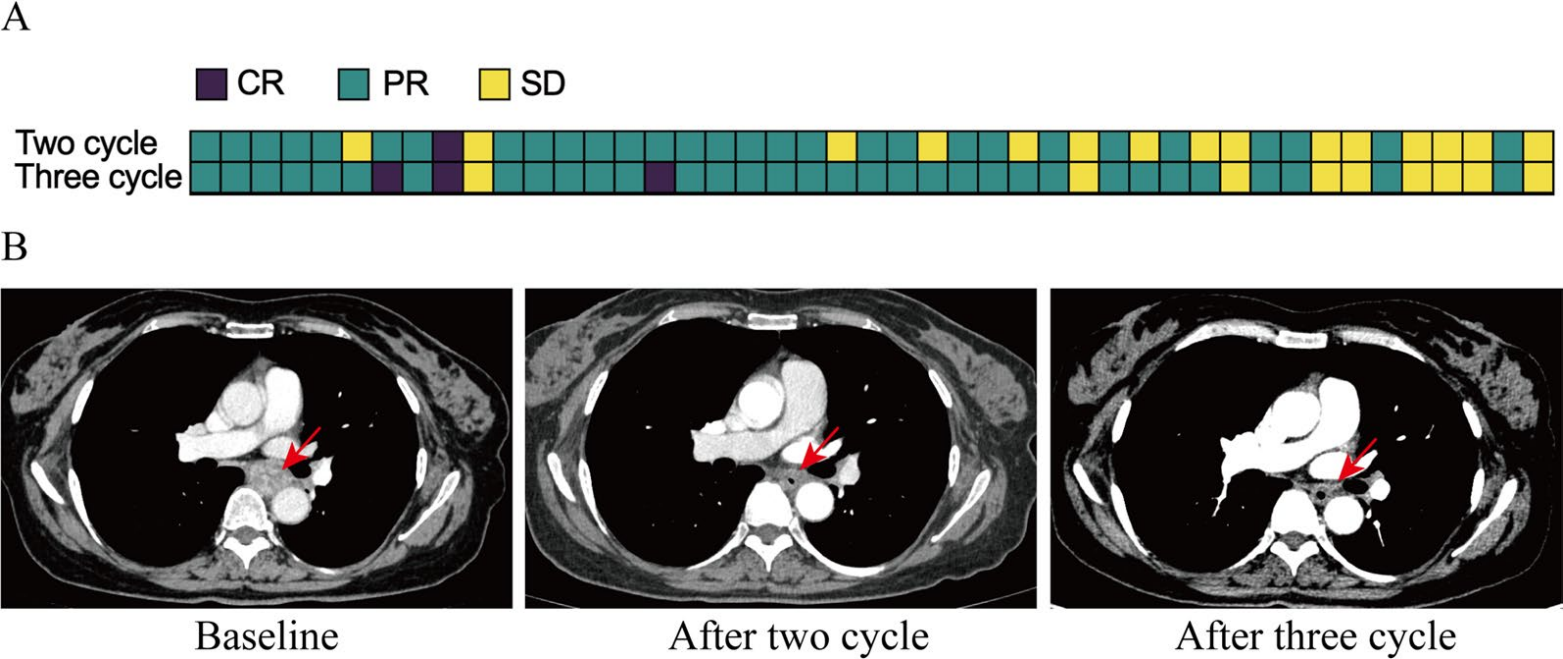
Clinical response to neoadjuvant immunochemotherapy. **A** Response assessment with computerized tomography (CT) before the initiation of treatment and after two and three cycles of neoadjuvant immunochemotherapy. **B** Representative CT scans of patient who achieved partial response (PR) following the second cycle and further achieved complete response (CR) after the third cycle treatment. Red arrow indicates the local tumor lesion. CR: complete response; PR: partial response; SD: stable disease

Among the 42 patients who underwent surgery, postoperative pathological results showed median tumor regression of 90% (range, 10–100%). A total of 27 (64.3%; 95% CI, 48–78.4%) patients had an MPR in the primary tumor. pCR was achieved in 14 (33.3%; 95% CI 19.6–49.5%) cases, of whom 13 (92.2%) had no residual tumor in either primary tissue or lymph node (Fig. 2). In terms of downstaging, 34 (81.0%; 95% CI 65.9–91.4%) patients were observed to have T-downstaging, and 32 (76.2%; 95% CI 60.5–87.9%) patients had N-downstaging after three cycles of neoadjuvant treatment. Radiographic response was not entirely concordant with pathological response. Eleven had PR according to CT but were found to have a pCR. One of the SD patients turned out to have a pCR (Fig. 1B, C).

Surgical tissues obtained from pCR or MPR patients were infiltrated by a large number of neutrophils and macrophages, fibrosis, and cholesterol clefts. Besides, we also observed classical TLSs, which have been reported to be associated with a favorable prognosis after immunotherapy [26, 27] (Fig. 1D).

### Efficacy and safety of two and three cycles of neoadjuvant treatment

CT and safety assessments were conducted before the initiation of treatment and after the second and third courses of neoadjuvant immunochemotherapy to compare the efficacy and safety of two and three treatment cycles. Of the 45 patients who had received three cycles of neoadjuvant treatment, two and three courses of treatment led to CR in 2.2% (1/45) versus 6.7% (3/45) (*P* = *0.616*), PR in 64.4% (29/45) versus 73.3% (33/45) (*P* = 0.503), and SD in 33.3% (15/45) versus 20.0% (9/45) (*P* = 0.167) of patients, with ORRs of 66.7% and 80.0% (*P* = 0.245). 6.9% (2/29) of patients who obtained PR after the second course of treatment achieved CR when the third course of treatment was completed (Fig. 2). 40.0% (6/15) of patients with SD after the second course improved to PR after an additional treatment cycle. In addition, three treatment cycles elicited a significantly higher rate of T down-staging than two (84.4% vs. 62.2%, *P* = 0.03). Three treatment cycles did not significantly increase TRAEs compared with two cycles (Additional file 1: Fig. S2).

### Survival

**Fig. 3:**
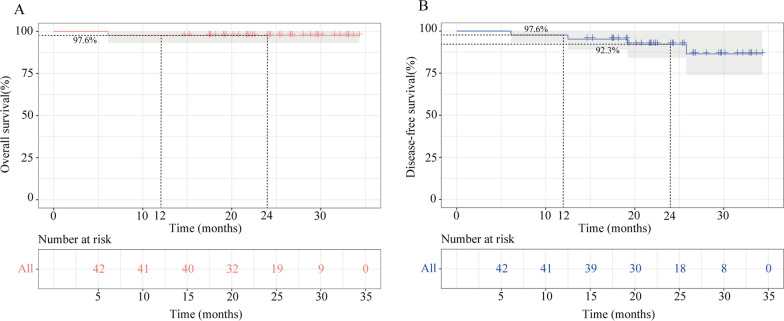
Survival of the patients who received surgery. **A** Overall survival. **B** Disease-free survival

The median follow-up was 24.3 months (range, 6.1–34.3 months) (database cutoff: May 12, 2023). The 1-year DFS and OS rates of the patients who received surgery were both 97.6%, and the 2-year DFS and OS rates were 92.3% and 97.6%, respectively. (Fig. 3). Three patients developed local recurrence or distant metastasis, wherein one experienced disease recurrence 12.5 months after surgery, and two developed metastasis at 19.2 and 25.8 months, respectively. One patient died 6 months after surgery due to cardiovascular diseases.

### Biomarker analyses

PD-L1 expression in pretreatment biopsies was measured in 34 patients. Four of 11 pCR and four of 23 non-pCR patients had a CPS of ≥ 1. No significant difference in PD-L1 expression was found between the pCR and non-pCR cases (*P* = 0.388). There was also no difference in the level of PD-L1 expression between groups stratified by T downstage or N downstage (Additional file 1: Table S2).

TMB is a biomarker for immunotherapy efficacy in multiple solid tumors. A total of 30 cases had adequate pretreatment specimens for NGS. We observed an average of 4.136 ± 1.431 mutations per patient. The most frequent driver mutations were *TP53*, *CDKN2A*, *CCND1*, *FGF19*, *FGF4*, and *CDKN2B* (Additional file 1: Fig. S3A). The frequency of the above genes was similar among the patients with pCR and non-pCR. No statistically significant difference in TMB levels was found between the patients with pCR and non-pCR (*P* = 0.308) (Additional file 1: Fig. S3B). No difference was noted in TMB levels between patients stratified by downstage (Additional file 1: Fig. S3C, Fig. 2D). Consistently, when patients were stratified by MPR and non-MPR, TMB levels were still similar between these two groups (Additional file 1: Fig. S4).

Annotated tissue specimens collected from 40 patients before neoadjuvant treatment and during surgery were subjected to multiplex immunofluorescence to get a glimpse of their TIME. Depending on sample quality, the TIME of tumor center and tumor stromal area in the pretreatment was analyzed in 35 and 40 patients, respectively. The densities of CD8^+^, TAMs (M1 and M2), and CD56^bright^ NK cells at baseline were similar between patients with pCR and those without pCR, either in tumor parenchyma or stroma. But the infiltration of CD56^dim^ NK cells in both the stroma and tumor parenchyma were significantly more abundant in the pCR group than in the non-pCR group (tumor parenchyma, *P* = 0.049; stroma, 0.012) (Additional file 1: Fig. S5A, S5B). When patients were stratified by MPR, the difference in the infiltration of CD56^dim^ NK cells in the stroma remained significant (Additional file 1: Fig. S6). In addition, tumor microenvironment change during neoadjuvant treatment was assessed by comparing the TIME of tumor stromal area in the biopsies collected before and after treatment in 40 patients. The abundance of infiltrating NK^dim^ cells in the stroma of tumor specimens significantly decreased after neoadjuvant immunochemotherapy in patients with pCR (*P* = 0.001). In contrast, no difference was found in the infiltration of this immune subset in the stroma of patients with non-pCR (Additional file 1: Fig. S7). The infiltration of CD8^+^, TAMs (M1 and M2), and CD56^bright^ NK cells before and after treatment were similar in both patients with pCR and those without pCR. Similar results were observed when TIME change was compared between MPR and non-MPR patients (Additional file 1: Fig. S8). Besides, the analysis of H&E- and immuno-stained surgical tumor tissues revealed a numerically higher density of TLSs in the pCR group compared to the non-pCR group (median 0.64 vs. 0.45/mm^2^, *P* = 0.351), which became statistically significant when comparing MPR and non-mPR (median 0.67 vs. 0.26/mm^2^, *P* = 0.002) (Additional file 1: Fig. S9).

## Discussion

In this phase II trial conducted in 47 patients with locally advanced ESCC, the pCR and MPR were 33.3% and 64.3%, respectively, and the ORR was 80.0%. Forty-two patients received surgery, and R0 resection was achieved in 100% of patients having undergone surgery. Three treatment cycles elicited a significantly higher rate of T down-staging than two (84.4% vs. 62.2%) without a significant increase in TRAEs. The most common TRAEs were grade 1–2, and no surgical delay was reported. With a median follow-up of 24.3 months, the 1-year DFS and OS rates were both 97.6%, and the 2-year DFS and OS rates were 92.3% and 97.6%, respectively. The density of CD56^dim^ NK cells in the pretreatment tissues was significantly higher in the pCR group than in the non-pCR group. While the density of TLSs in the posttreatment tissues was numerically higher in the pCR group, and this difference became statistically significant when comparing patients with MPR to those with non-MPR. No difference was found in PD-L1 expression and TMB levels between pretreatment specimens of the pCR and non-pCR patients.

Neoadjuvant therapy is recommended in many cancers to achieve tumor downstaging and improve the curative rate. However, different cycles of neoadjuvant treatment could influence the prognosis and the quality of perioperative life. In a randomized phase II study [28], three courses of preoperative chemotherapy led to a better response without increasing TRAEs or morbidity than two courses in ESCC. For neoadjuvant immunochemotherapy, all the available clinical trials have focused on the effects of two-cycle regimens, which could be efficiently limited. In our previous pilot study [21], three cycles of neoadjuvant immunochemotherapy was safe and feasible, which was further confirmed in this phase II trial.

The toxicity of intensive cycles of camrelizumab plus chemotherapy was tolerated. Most of the TRAEs were grade 1–2, which was similar to previous data of two treatment cycles [15, 16]. RCCEP was found in 28 (59.6%) patients. The incidence was higher than those of the two treatment cycles (26.1–39.1%) [15, 16]. This difference may have resulted from the additional course of camrelizumab. In addition, more than half of patients experienced leukopenia after receiving neoadjuvant immunochemotherapy or chemoradiotherapy, according to previous reports [6, 16]. Severe leukopenia can even lead to dose reduction or termination of treatment. In our study, there was only a low frequency (6.4%) of leukopenia. The difference could be attributed to the following reasons. First, we prophylactically used G-CSF after each course of chemotherapy treatment. Second, platinum was replaced with capecitabine in our regimen. Capecitabine is an oral drug and can be converted to fluorouracil [29]. The combination of capecitabine with paclitaxel exhibited similar efficacy but lower toxicity compared with platinum-based regimens in breast cancer and head and neck squamous cell carcinoma [30–32]. The toxicity of this drug is relatively low, which might render it a suitable candidate for combination with PD-1 blockade. Overall, the toxicity of our neoadjuvant regimen was manageable and worthy of promotion.

For surgery completion, R0 resection was achieved in 100% of patients who underwent surgery, which was consistent with that of other two-cycle regimens (96.3–100%) [16, 33]. The volumes of lymph node dissection far exceeded those of other two-cycle camrelizumab treatment [16]. It seems that one additional course of immunotherapy would not increase the difficulty in conducting surgery and lymph node dissection. With regard to complications, anastomotic leakage was the most frequent complication, with an incidence of 19.0%, which was in the normal range compared with surgery alone (15% to 20%) [34]. Moreover, the time of operation duration and patient hospital stays were not prolonged. No perioperative deaths were reported in our cohort. These results suggested that the intensive-cycle regimen was feasible.

In this study, the pCR rate was 33.3%, similar to the results from other immunochemotherapy trials of ESCC. Taking an intensive-cycle regimen does not seem to impact the pCR (data from two-cycle immunochemotherapy studies: 25–35.3%) [16, 35–37]. Whereas, CT assessment conducted at treatment milestones (before and after the second and third course of neoadjuvant therapy) indicated that three treatment cycles elicited a significantly higher rate of T down-staging than two (84.4% vs. 62.2%, *P* = 0.03), without increasing TRAEs, suggesting the feasibility and safety of three cycles of immunochemotherapy to increase tumor regression. These results were consistent with data from locally advanced lung cancers [17, 38].

Furthermore, our study found that 2.4% (1/42) of patients developed local recurrence and 4.8% (2/42) experienced distant metastasis at a median follow-up time of 24.3 months after surgery, which were numerically lower than the respective rates of 20% (4/20) and 10% (2/20) observed in patients who received two cycles of neoadjuvant PD-1 blockade plus chemotherapy at a median follow-up time of 13.5 months [16]. Both the 1-year OS (97.6% vs. ~ 90.0%) and DFS (97.6% vs. ~ 80.0%) were numerically higher than those with two-cycle immunochemotherapy regimens [16, 36]. The potential explanation for these data was that except for the advantage of increasing tumor shrinkage, intensive cycles of immunochemotherapy might exert longer-term antitumor activity, thereby inducing longer-term tumor regression and eradicating micrometastases. Follow-up is ongoing, and long-term survival data will be released in the future.

The pathological response was significantly predictive of prognosis [39]. It is essential to explore biomarkers to identify patients who might benefit from the treatment. PD-L1 and TMB were the most commonly investigated biomarkers. Our study found that the level of PD-L1 expression and TMB at baseline had poor correlations with the pathological response, which was consistent with previous studies that PD-L1 and TMB failed to precisely predict the efficacy of neoadjuvant immunotherapy [40, 41]. Instead, CD56^*dim*^ cells were found at a higher density in the pretreatment biopsy of responders. This observation was consistent with our previous finding [21]. CD56 cells are the major subtype of NK cells and the primary force of innate immunity for anti-tumor response [42]. Moreover, we observed a decrease in the density of NK^*dim*^ cells in the stroma after immunotherapy in responders but not in non-responders. The decrease in the infiltration of CD56 cells in the stroma might have been attributed to the fact that PD-1 blockade could induce the mobilization of more abundant NK cells to infiltrate from the stroma to the parenchyma. Previous work in melanoma supported that immune cells infiltrated from the tumor edge and gradually infiltrated to the core of the tumor upon immunotherapy treatment [43]. Furthermore, TLSs were found to be more abundant in surgical tissues from pCR or MPR patients than those from non-pCR or non-MPR patients, which was consistent with previous reports linking TLSs to a favorable prognosis following immunotherapy [26, 27].

Molecular genetic analyses demonstrated multiple genetic abnormalities in ESCC. Our study found some specific driver mutations, including *CCND1*, *FGF19*, and *FGF4*. These genes were located in 11q13, which has been considered the most frequently amplified locus in ESCC and is related to the development of ESCC [44]. However, all the driver mutations failed to predict the response to immunotherapy. This might have resulted from the complex genomic context in locally advanced ESCC. It would be difficult to predict prognosis with a single gene.

To summarize, intensive cycles of neoadjuvant chemotherapy combined with camrelizumab demonstrated favorable efficacy and acceptable toxicity, particularly an encouraging 1-year DFS and OS. The abundance of CD56^*dim*^ NK cells in the pretreatment tumor tissue might be a potential biomarker to predict the efficacy of immunotherapy in locally advanced ESCC. The follow-up of this study is still ongoing, and the long-term survival data will be released in the future. Due to the limited sample size and the single-arm manner of the study, randomized controlled trials with larger sample sizes are needed to confirm our findings.

## Supplementary Information

**Additional file 1. **Additional Tables and Figures.

## Data Availability

Data are available upon reasonable request. All data relevant to this study are included in the article or uploaded as Supplemental information.

## Abbreviations

ESCC: Esophageal squamous cell carcinoma
TIME: Tumor immune microenvironment
TMB: Tumor mutational burden
PD-1: Programmed death-1
PD-L1: Programmed death-ligand 1
pCR: Complete pathological response
MPR: Major pathological response
EUS: Endoscopic ultrasonography
CT: Computerized tomography
TRAEs: Treatment-related adverse events
H&E: Hematoxylin and eosin
CPS: Combined positive score
mIF: Multiplex immunofluorescence
CAP: College of American Pathologists
CLIA: Clinical Laboratory Improvement Amendments
NGS: Next-generation sequencing
FFPE: Formalin-fixed paraffin-embedded
SNVs: Single nucleotide variants
Indels: Insertions/deletions
LN: Lymph node
SD: Stable disease
PR: Partial response
95% CI: 95% Confidence interval
