# Supplementary material for: Intensive cycles of neoadjuvant camrelizumab combined with chemotherapy in locally advanced esophageal squamous cell carcinoma: a single-arm, phase II trial
Source: J Transl Med. 2023 Jun 24;21:411. doi: 10.1186/s12967-023-04273-6 (PMC10290378; doi:10.1186/s12967-023-04273-6)
Supplement: Supplementary file 1 — Additional file 1. Additional Tables and Figures. [file 12967_2023_4273_MOESM1_ESM.docx]

**Table S1. Summary of surgery-related adverse events**

| **Complication** | **No. of patients** |
| --- | --- |
| anastomotic leakage | 8 (19.09%) |
| chylothorax | 1 (2.4%) |
| bleeding | 0 (0%) |
| Immune-related myocarditis | 0 (0%) |
| Immune-related nephritis | 0 (0%) |
| Immune-related hepatitis | 0 (0%) |
| Immune-related pneumonia | 0 (0%) |
| Immune-related hyperthyroidism | 0 (0%) |
| Immune-related hypothyroidism | 0 (0%) |
| Death within 90 days | 0 (0%) |

**Table S2. The association between PD-L1 expression and treatment response**

|  | **PD-L1**  **(CPS<1)** | **PD-L1**  **(CPS≥1)** | **Total** | **P Value** |
| --- | --- | --- | --- | --- |
| **pCR** | 7 | 4 | 11 | 0.388 |
| **Non-pCR** | 19 | 4 | 23 |  |
| **Total** | 26 | 8 | 34 |  |

|  | **PD-L1**  **(CPS<1)** | **PD-L1**  **(CPS≥1)** | **Total** | **P Value** |
| --- | --- | --- | --- | --- |
| **N downstage** | 19 | 6 | 25 | 0．999 |
| **N non-downstage** | 7 | 2 | 9 |  |
| **Total** | 26 | 8 | 34 |  |

|  | **PD-L1**  **(CPS<1)** | **PD-L1**  **(CPS≥1)** | **Total** | **P Value** |
| --- | --- | --- | --- | --- |
| **T downstage** | 21 | 7 | 28 | 0.999 |
| **T non-downstage** | 5 | 1 | 6 |  |
| **Total** | 26 | 8 | 34 |  |

**Abbreviations:** pCR, complete pathologic response. PD-L1, programmed death ligand 1. CPS, combined positive score (CPS), defined as the number of PD-L1-stained tumor and immune cells divided by the total number of viable tumor cells and multiplied by 100


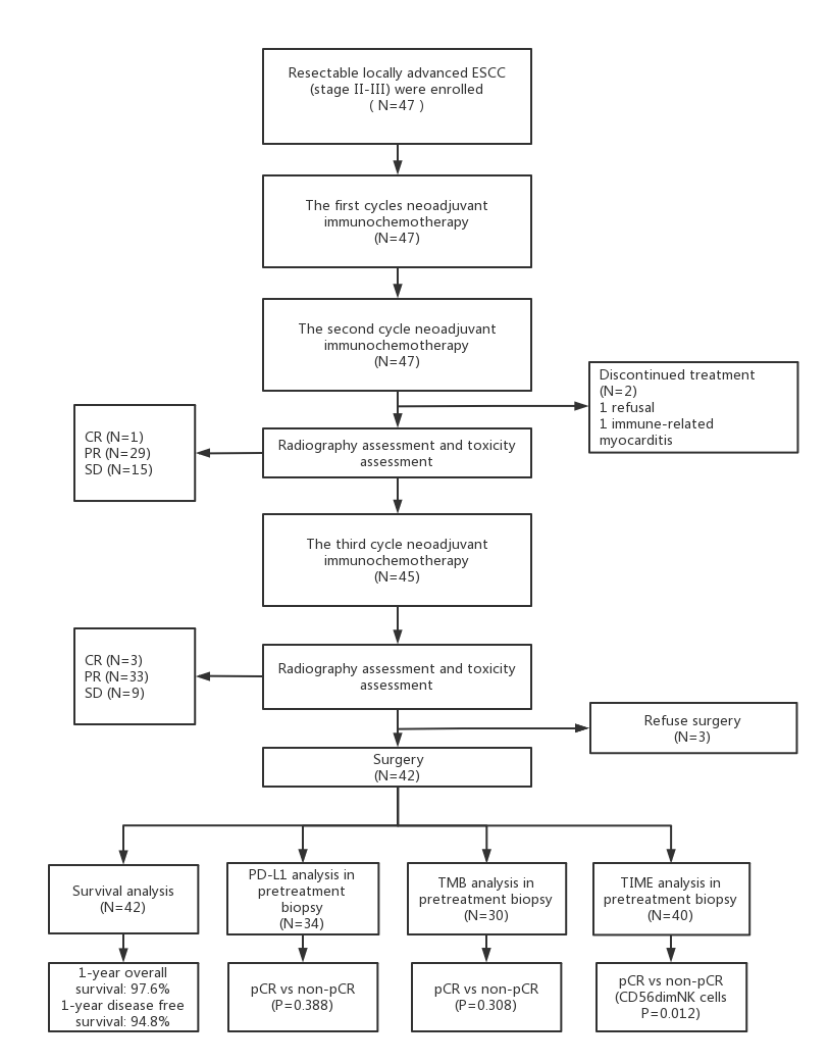


**Figure S1 Flow chart of the study**

Forty-seven patients were enrolled and received neoadjuvant immunochemotherapy. Hence the intention-to-treat population consisted of 47 patients, in which the efficacy and safety analyses were performed. Five patients discontinued treatment due to the reasons indicated. Forty-two patients received surgery.


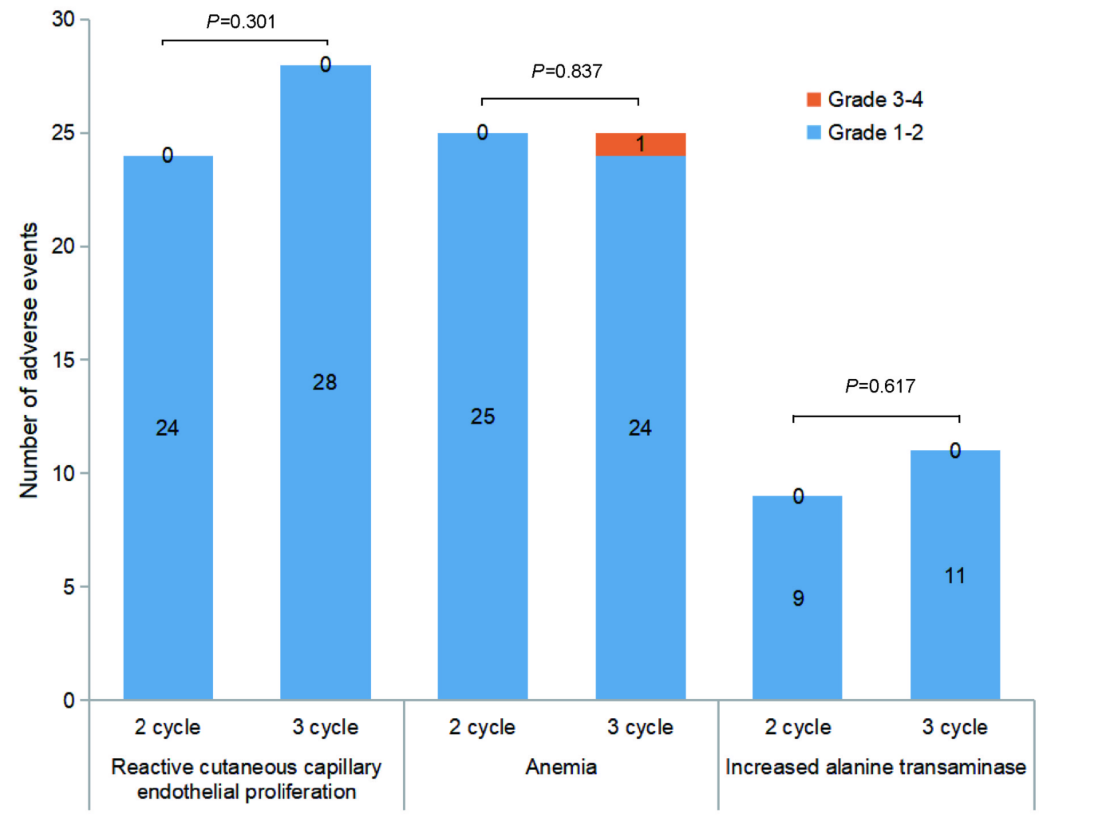


**Figure S2** **Adverse events with an increase in number or grade during the 3rd cycle of treatment**

Of the 47 patients who received two cycles of immunotherapy combined with chemotherapy, two discontinued treatment after the second cycle, with one patient experiencing immune-related myocarditis and the other withdrawing informed consent. As a result, a total of 45 patients completed the entire course of treatment, receiving three cycles of immunotherapy combined with chemotherapy. The incidence of most complications remained unchanged between the 2 and 3 cycles of immunochemotherapy. The 3rd cycle of treatment only caused a slight increase in the number or grade of three complications.

**
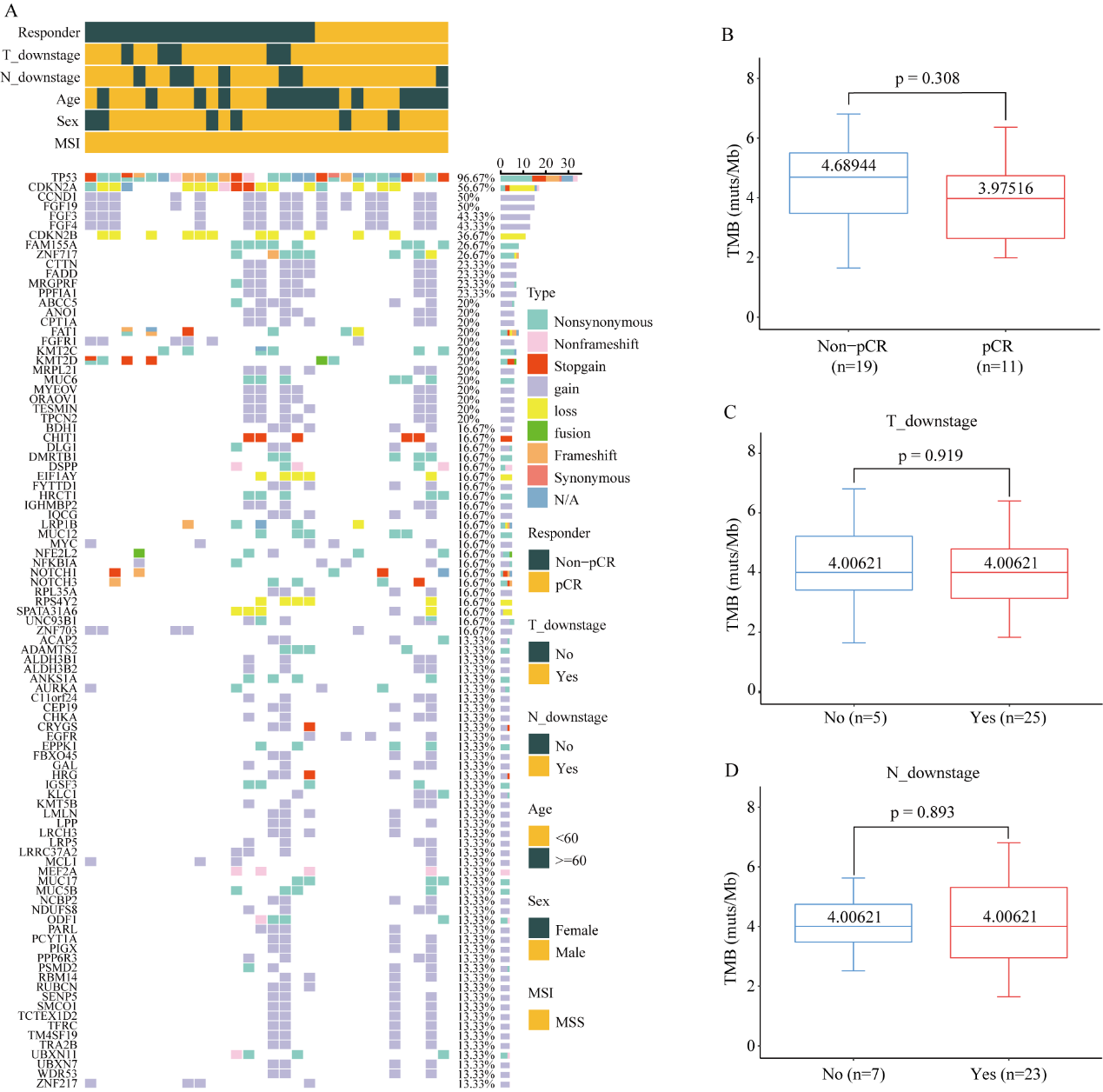
**

**Figure S3.** **Correlation between gene variation and pathological response.** (A) Pretreatment tissue biopsies from 30 ESCC patients were subjected to genomic profiling using a 733-gene NGS panel or whole-exome sequencing (WES). Variants with a frequency of ≥ 10% were shown in the chart. (B) Tumor mutational burden among the patients with pCR and without pCR (non-pCR). (C) Tumor mutational burden (TMB) among patients with or without T downstage. (D) Tumor mutational burden among patients with or without N downstage. pCR, complete pathologic response. TMB, tumor mutational burden. TMB levels were adjusted using the TMB correlation factor of the NGS 733-gene panel with WES. MSI, microsatellite instability.

**
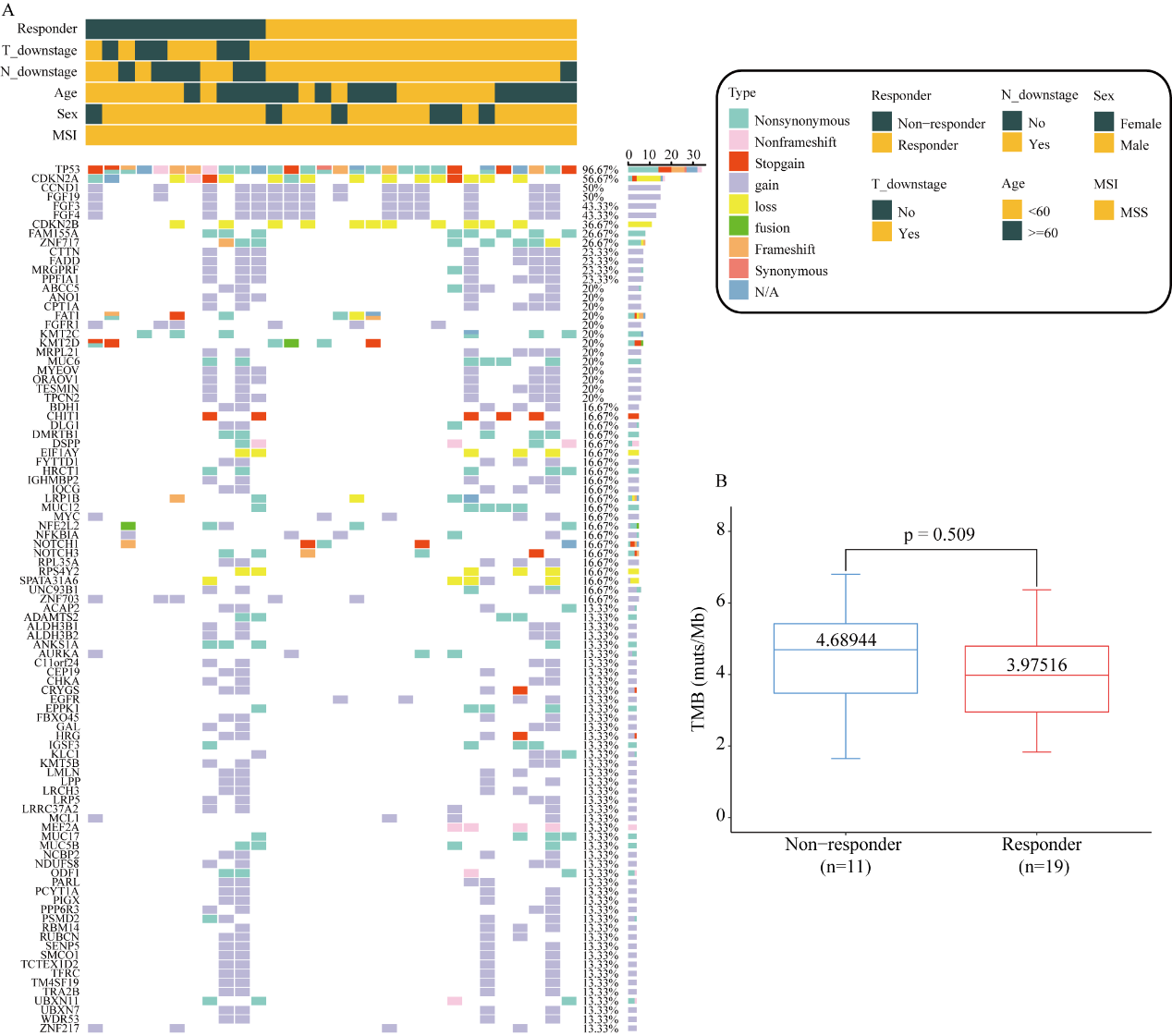
**

**Figure S4. Correlation between gene variation and pathological response.** (A) Pretreatment tissue biopsies from 30 ESCC patients were subjected to genomic profiling using a 733-gene NGS panel or whole-exome sequencing (WES). Variants with a frequency of ≥ 10% were shown in the chart. (B) Tumor mutational burden (TMB) among the responders and non-responders. Patients with pCR or MPR were collectively defined as responders. Patients with more than 10% residual tumor in the primary tumor tissue were regarded as non-responders. pCR, complete pathologic response; MPR, major pathologic response. TMB levels were adjusted using the TMB correlation factor of the 733-gene NGS panel with WES. MSI, microsatellite instability.


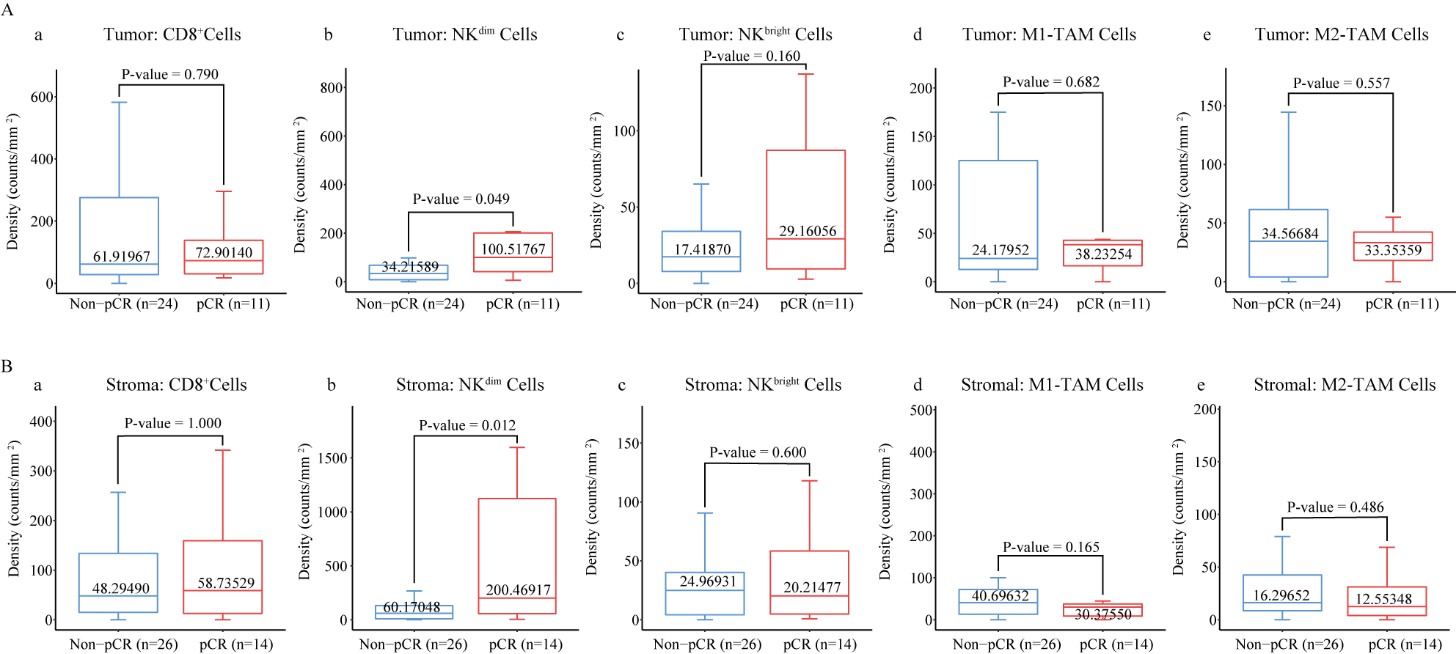


**Figure S5.** **Correlation between pathological response and TIME in the pretreatment tissue biopsy.** (A) Correlation between pathological response and the infiltration of immune cell subsets, including CD8^+^ cells (a), NK^dim^ cells (b), NK^bright^ cells (c), M1-TAM cells (d), and M2-TAM cells (e) in the tumor area (n=35); (B) Correlation between pathological response and the infiltration of immune cell subsets, including CD8^+^ cells (a), NK^dim^ cells (b), NK^bright^ cells (c), M1-TAM cells (d), and M2-TAM cells (e) in the tumor stroma area (n=40). Density was defined as the number of stained cells per square millimeter. TIME, tumor immune microenvironment. pCR, complete pathological response.

**
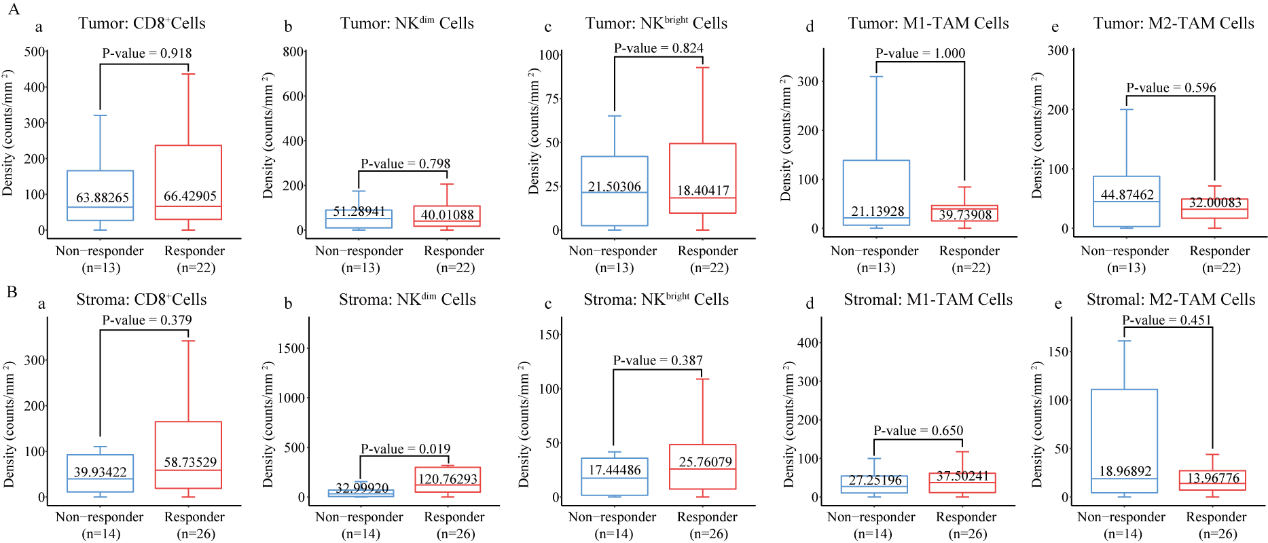
**

**Figure S6.** **Correlation between pathological response and TIME in the pretreatment tissue biopsy.** (A) Correlation between pathological response and the infiltration of immune cell subsets, including CD8^+^ cells (a), NK^dim^ cells (b), NK^bright^ cells (c), M1-TAM cells (d), and M2-TAM cells (e) in the tumor area (n=35). (B) Correlation between pathological response and the infiltration of immune cell subsets, including CD8^+^ cells (a), NK^dim^ cells (b), NK^bright^ cells (c), M1-TAM cells (d), and M2-TAM cells (e) in the tumor stroma area (n=40). Density was defined as the number of stained cells per square millimeter. TIME, tumor immune microenvironment. Patients with no more than 10% residual tumor in the primary tumor tissue (MPR+pCR) were defined as responders. Patients with more than 10% residual tumor in the primary tumor tissue were regarded as non-responders. pCR, complete pathologic response; MPR, major pathologic response.


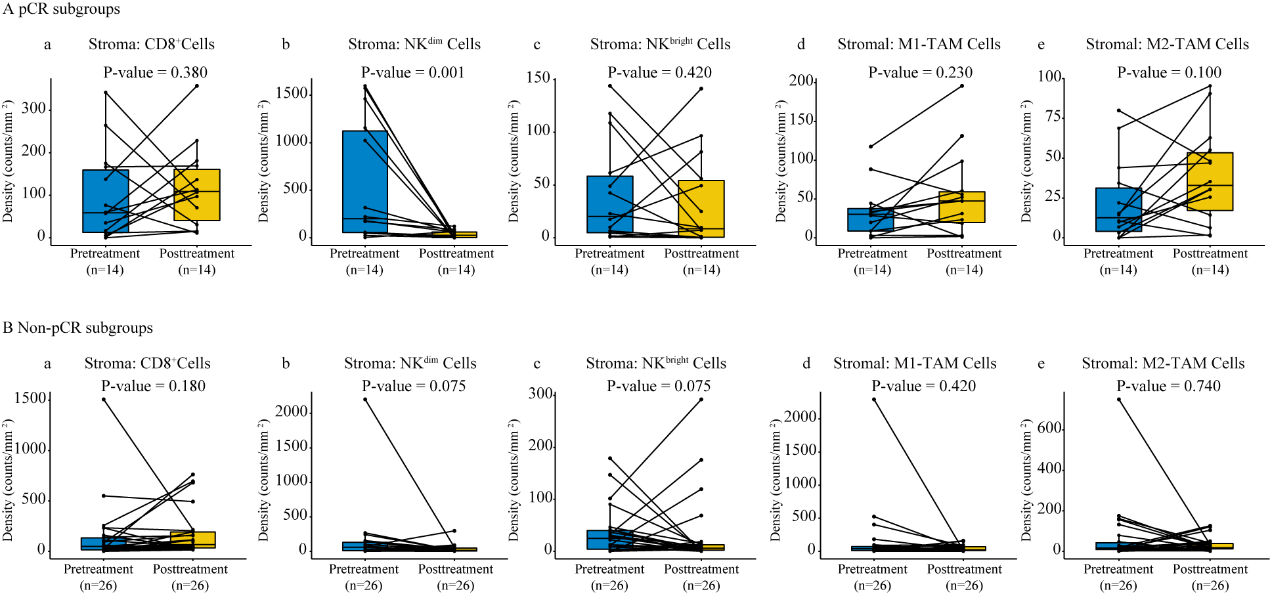


**Figure S7. Correlation between pathological response and the change in the TIME before and after neoadjuvant therapy.** (A) Infiltration of immune cell subsets, including CD8^+^ cells (a), NK^dim^ cells (b), NK^bright^ cells (c), M1-TAM cells (d), and M2-TAM cells (e) in the tumor stroma of pretreatment and posttreatment tissue specimens from patients with pCR (n=14). (B) Infiltration of immune cell subsets, including CD8^+^ cells (a), NK^dim^ cells (b), NK^bright^ cells (c), M1-TAM cells (d), and M2-TAM cells (e) in the tumor stroma of pretreatment and posttreatment tissue specimens from patients with non-pCR (n=26). Density was defined as the number of stained cells per square millimeter. TIME, tumor immune microenvironment. pCR, complete pathological response.

**
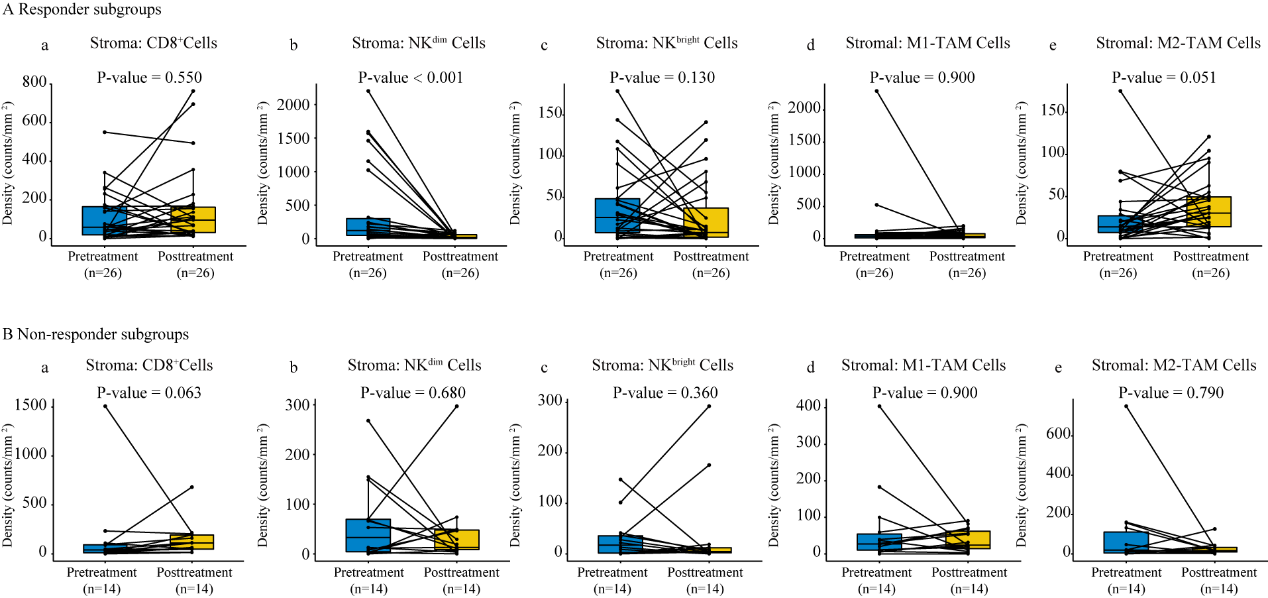
**

**Figure S8. Correlation between pathological response and the change in the TIME before and after neoadjuvant therapy.** (A) Infiltration of immune cell subsets, including CD8^+^ cells (a), NK^dim^ cells (b), NK^bright^ cells (c), M1-TAM cells (d), and M2-TAM cells (e) in the tumor stroma of pretreatment and posttreatment tissue specimens from responders (n=14). (B) Infiltration of immune cell subsets, including CD8^+^ cells (a), NK^dim^ cells (b), NK^bright^ cells (c), M1-TAM cells (d), and M2-TAM cells (e) in the tumor stroma of pretreatment and posttreatment tissue specimens from non-responders (n=26). Density was defined as the number of stained cells per square millimeter. TIME, tumor immune microenvironment. Patients with no more than 10% residual tumor in the primary tumor tissue (MPR+pCR) were defined as responders. Patients with more than 10% residual tumor in the primary tumor tissue were regarded as non-responders. pCR, complete pathologic response; MPR, major pathologic response.


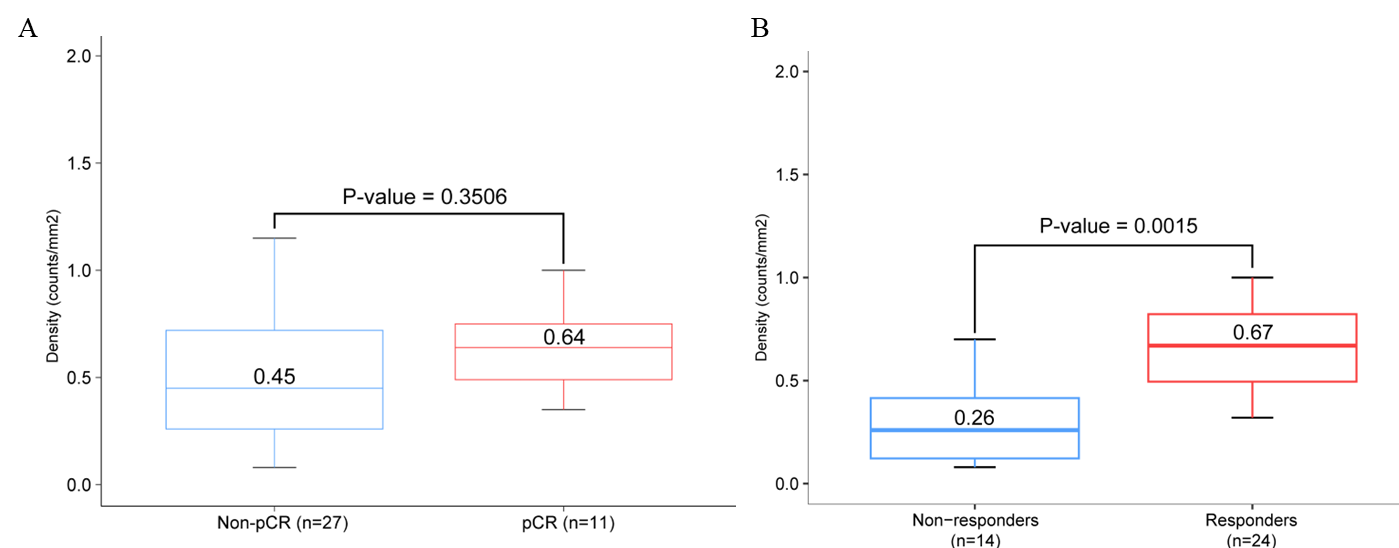


**Figure S9. Correlation between pathological response and the formation of tertiary lymphoid structures after neoadjuvant therapy.** Tertiary lymphoid structures were visualized by hematoxylin and eosin-staining and immunostaining for CD3 and CD20. (A) The density of tertiary lymphoid structures in the resected tumor specimens from pCR and non-pCR patients. (B) The density of tertiary lymphoid structures in the resected tumor specimens from responders and non-responders. Patients with no more than 10% residual tumor in the primary tumor tissue (MPR+pCR) were defined as responders. Patients with more than 10% residual tumor in the primary tumor tissue were considered non-responders. pCR, complete pathologic response; MPR, major pathologic response.
